# Risk factors and diagnostic prediction models for papillary thyroid carcinoma

**DOI:** 10.3389/fendo.2022.938008

**Published:** 2022-09-05

**Authors:** Xiaowen Zhang, Yuyang Ze, Jianfeng Sang, Xianbiao Shi, Yan Bi, Shanmei Shen, Xinlin Zhang, Dalong Zhu

**Affiliations:** ^1^ Department of Endocrinology and Metabolism, Endocrine and Metabolic Disease Medical Center, Nanjing University Medical School Affiliated Drum Tower Hospital, Nanjing, China; ^2^ Department of Endocrinology and Metabolism, The Fifth People’s Hospital of Suzhou Wujiang, Suzhou, China; ^3^ Department of Thyroid Surgery, Nanjing University Medical School Affiliated Drum Tower Hospital, Nanjing, China; ^4^ Department of Cardiology, Nanjing University Medical School Affiliated Drum Tower Hospital, Nanjing, China

**Keywords:** papillary thyroid carcinoma, logistic regression analysis, back propagation neural network, diagnostic prediction, Bethesda category

## Abstract

Thyroid nodules (TNs) represent a common scenario. More accurate pre-operative diagnosis of malignancy has become an overriding concern. This study incorporated demographic, serological, ultrasound, and biopsy data and aimed to compare a new diagnostic prediction model based on Back Propagation Neural Network (BPNN) with multivariate logistic regression model, to guide the decision of surgery. Records of 2,090 patients with TNs who underwent thyroid surgery were retrospectively reviewed. Multivariate logistic regression analysis indicated that Bethesda category (OR=1.90, P<0.001), TIRADS (OR=2.55, P<0.001), age (OR=0.97, P=0.002), nodule size (OR=0.53, P<0.001), and serum levels of Tg (OR=0.994, P=0.004) and HDL-C (OR=0.23, P=0.001) were statistically significant independent differentiators for patients with PTC and benign nodules. Both BPNN and regression models showed good accuracy in differentiating PTC from benign nodules (area under the curve [AUC], 0.948 and 0.924, respectively). Notably, the BPNN model showed a higher specificity (88.3% vs. 73.9%) and negative predictive value (83.7% vs. 45.8%) than the regression model, while the sensitivity (93.1% vs. 93.9%) was similar between two models. Stratified analysis based on Bethesda indeterminate cytology categories showed similar findings. Therefore, BPNN and regression models based on a combination of demographic, serological, ultrasound, and biopsy data, all of which were readily available in routine clinical practice, might help guide the decision of surgery for TNs.

## 1 Introduction

The incidence of thyroid cancer has increased remarkably worldwide in recent years (1), with a more than 3-fold increase from 1974 to 2013 in the United States (2, 3). Thyroid cancer has also become the fastest-growing cancer among women at the beginning of this century in China (4). The sharp increase in the incidence of thyroid nodules (TNs) (5), although most of which are benign, adds to the burden of health system (6). Accurate pre-operative diagnosis of potentially malignant tumors is warranted.

Most guidelines recommend ultrasound as the first diagnostic approach for TNs. Thyroid Imaging Reporting and Data Systems (TI-RADS) are widely used to guide clinical practice. Recommendations for diagnostic fine-needle aspiration biopsy (FNAB) of TNs are based on sonographic features combined with nodule sizes. Following biopsy, the Bethesda System for Reporting Thyroid Cytopathology is used worldwide to classify FNAB cytology findings and determine whether surgery is needed. However, there are limitations with the categorical Bethesda system. Bethesda III and IV cytology diagnoses, known as indeterminate, comprise approximately 30% of FNAB results (7); and management of this group of patients varies widely from clinical observation, ultrasound follow up, repeat FNAB, molecular test to thyroid surgery. Overdiagnosis and surgery of thyroid cancer are common in clinical practice. Therefore, developing a systematic method to differentiate patients with papillary thyroid carcinomas (PTC) from benign nodules is important to guide surgery decision.

A back propagation neural network (BPNN) model is a kind of classical nonlinear artificial neural network (ANN) model based on the Deepest-Descent technique. When provided with sufficient hidden units, it will repeatedly adjust the weights of connections in the network and minimize the error of nonlinear functions between the actual and expected output values. First proposed in 1986 (8), BPNN has been applied to help clinical diagnosis, imaging, and prognosis prediction (9). Multiple studies have focused on using ultrasound images of TNs in deep convolutional neural networks (10, 11). However, to our knowledge, no study has yet combined demographic, serological, ultrasound, and biopsy data into one model. In this study, we aimed to construct a new diagnostic prediction model of PTC based on BPNN and compare it with conventional multivariate logistic regression model.

## 2 Materials and methods

### 2.1 Human subjects

This study protocol was approved by the Ethics Committee of Nanjing Drum Tower Hospital Institutional Review Board, and consent was waived for this retrospective study. We conducted a retrospective analysis of consecutive patients who underwent thyroid surgery due to TNs in the Department of Thyroid Surgery of Nanjing Drum Tower Hospital from January 2018 to January 2021. The inclusion criteria were as follows (1): aged 18 years or older (2), undergoing thyroidectomy (total thyroidectomy, unilateral and isthmic excision, double-lobed subtotal excision, etc.) (3), post-operative pathologic diagnosis was PTC or benign TNs. The exclusion criteria were as follows (1): history of other malignancies (2), previous thyroid surgery (3), incomplete clinical data (4), liver failure, renal failure, or severe infection in the last 3 months.

### 2.2 Demographic data, serum hormone and biochemical analysis

Baseline demographic characteristics including age, gender, family history of thyroid tumor, history of radiation, blood pressure, and body mass index (BMI) were collected. Serum thyroid-stimulating hormone (TSH), free triiodothyronine (FT3), free thyroxine (FT4), thyroid autoantibodies [TAb; thyroid peroxidase antibody (TPOAb) and thyroglobulin antibody (TgAb)], and thyroglobulin (Tg) concentrations were detected by electrochemical luminescence assays with Cobas Eless 601 (Roche Diagnostics, Basel, Switzerland). The reference ranges of TSH, TPOAb, and TgAb were 0.27–4.2 mIU/L, 0–34 IU/ml, and 0–115 IU/ml, respectively, as provided by the manufacturer. Serum high-density lipoprotein cholesterol (HDL-C) levels were tested with standard enzymatic methods (Kyowa Medex Co., Ltd. Tokyo, Japan), and serum low-density lipoprotein cholesterol (LDL-C) levels were measured with selective melt method (Kyowa Medex Co., Ltd. Tokyo, Japan).

### 2.3 Thyroid ultrasonography

All patients underwent two rounds of thyroid ultrasound examinations before surgery. They first received one in the out-patient department, by a sonologist randomly determined by the clinic appointment system. When they were hospitalized in the Thyroid Surgery Department, they received their second ultrasound test, performed by two specified sonologists with over 10-year experience. The sonologists were unaware of the cytopathology and histopathology, as well as of laboratory results of the patients. Kwak-TIRADS criteria were applied to each nodule for categorization. When more than one nodule was present in the thyroid, the nodule with the highest TIRADS score was chosen for analysis. The radiology reports were extracted from clinical records.

### 2.4 Thyroid nodule pathology

The ultrasound-guided FNAB was conducted by a senior sonologist, and the FNAB cytology slides were examined according to the Bethesda System for Reporting Thyroid Cytopathology (7). The pathologists were blinded to the sonographic diagnosis of TNs. The FNAB results were classified into six categories, and Bethesda III to V cytopathology were categorized as indeterminate cytology (12).

### 2.5 Statistical analyses

Continuous variables were presented as means ± standard deviation and categorical variables as numbers with percentages. Differences between patients with benign nodules and PTC were analyzed using the independent sample t test for continuous variables, and χ 2 test for categorical variables. Logistic regression analysis was performed to identify differentiating variables for patients with benign nodules from PTC. To account for the risk of type 1 error due to multiple comparison, Bonferroni correction was applied with an adjusted P value threshold of 0.003 (0.05/16). Variables with statistical significance (P<0.003) in univariate analysis and those with a high clinical relevance were included in multivariable logistic regression analysis with a backward stepwise selection mode.

Ten patients receiving first-round ultrasound examinations by one sonologist were assigned to receive the second-round ultrasound test by the other one. Interobserver variability of ultrasound features obtained from two sonologists was investigated by using intraclass correlation coefficients. Intraclass correlation coefficients less than 0.40 indicate poor agreement; 0.41–0.75, moderate agreement; and 0.75 or greater, good agreement. SPSS v26.0 software (IBM Corp., Armonk, NY, United States) was used for these statistical analyses.

### 2.6 BPNN model

#### 2.6.1 Basic principles and parameter selection

ANN models are statistical models that simulate the cognitive processes of human brain, consisting of mess of neuron nodes. Compared with the traditional statistical methods or computer algorithms, it has good fault tolerance, highly non-linear, self-learning, self-organization, and self-adaptability (13). BPNN is one of the most widely used classical types of ANN. It includes an input layer, an output layer, and several hidden layers. Information propagates forward from the input layer to the hidden layer and the output layer. In each neuron, the information input from the previous layer is processed by the activation function and then inputted to the next layer. In each iteration, the weight coefficients of the nodes are modified using new data from the training data set, and the error is minimized after several iterations, as shown in **Figure S1**. A sigmoid function was used as the activation function to map the range of input values from (-∞, +∞) to the interval (0, 1). The number of iterations was 1,000 and the learning rate was 0.01 after several runs and adjustments.

#### 2.6.2 Input and output variables

Input variables were selected in the BPNN model according to the results of the univariate logistic regression analyses and the notable clinical relevance of family history of thyroid tumor and history of radiation exposure. The output variable was pathological results (benign TN was assigned to 1 and PTC to 2).

#### 2.6.3 Software implementation

The BPNN toolbox of MATLAB R2016b software (MathWorks Inc., Natick, Massachusetts, United States) was used to construct the BPNN model. The specific code is shown in supplemental file. The neural analysis was run with 70% of the cases (1,463 cases), randomly selected as the training set, and the remaining 30% (627 cases) as the prediction set, as previously reported (14). Our study conducted a conventional 3-layer BPNN, with an input layer, a hidden layer, and an output layer. The optimal number of nodes in the hidden layer of the BPNN was determined by repetitious data simulation, and 15 hidden neurons were adopted as the optimal case (**Table S1**). The differentiating performance of the BPNN model was assessed with parametric receiver operating characteristic curve (ROC) analysis.

#### 2.6.4 Evaluation of diagnostic performance

GraphPad Prism 8.2.1 software (GraphPad Software Inc., San Diego, California, United States) was used to plot ROC curves. We calculated the true positive (TP), false positive (FP), true negative (TN), false negative (FN) values, as well as sensitivity, specificity, Youden index, negative predictive value (NPV), and positive predictive value (PPV) for each model.

## 3 Results

### 3.1 Baseline characteristics of patients with PTC compared with benign nodules

**Figure 1:**
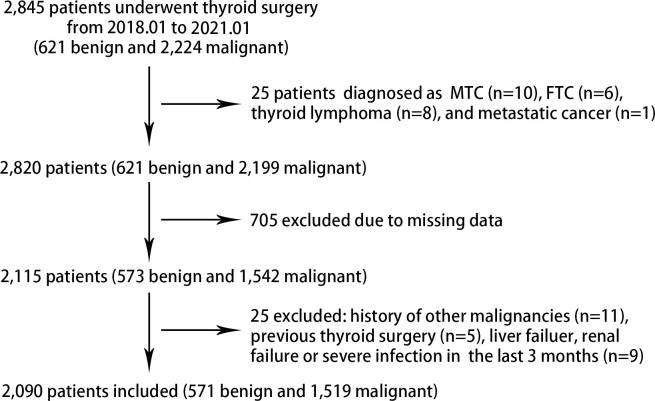
Flowchart of patient selection in the study. FTC, follicular thyroid carcinoma; MTC, medullary thyroid cancer.

A total of 2,090 patients (546 males and 1,544 females) with TNs were included in the analysis, among whom 571 were with benign TNs (thyroid cystic adenoma, hyperplastic nodular goiter, etc.) and 1,519 were with PTC (**Figure 1**). Baseline characteristics are shown in **Table 1**. Patients with PTC tended to be younger (43.2 ± 12.4 vs. 50.8 ± 13.1 years, P<0.001), and were more likely to have family history of thyroid tumor (4.9% vs. 2.8%; P=0.038) and radiation exposure (2.7% vs. 0.1%; P=0.011), as compared with those with benign nodules. Patients with PTC also showed a higher Bethesda category (4.96 ± 1.44 vs. 2.66 ± 1.53, P<0.001) and Kwak TIRADS score (5.11 ± 1.15 vs. 3.31 ± 0.66, P<0.001), but a lower nodule diameter (1.2 ± 0.89 vs. 3.24 ± 1.6, P<0.001).

**Table 1:** Baseline characteristics of TN patients based on nodule types.

Indicator	Total (N=2090)	Benign TN (N=571)	PTC (N=1519)	*P*
Gender				0.042
Male	546 (26.1)	131(22.9)	415 (27.3)	
Female	1544 (73.9)	440 (77.1)	1104 (77.7)	
Age (years)	45.28 ± 13.01	50.77 ± 13.09	43.22 ± 12.38	<0.001
BMI (kg/m^2^)	23.83 ± 3.45	23.85 ± 3.30	23.80 ± 3.51	0.892
History of Radiation				0.011
No	2044 (97.8)	566 (99.9)	1478 (97.3)	
Yes	46 (2.2)	5 (0.1)	41 (2.7)	
Family History				0.038
No	2000 (95.7)	555 (97.2)	1445 (95.1)	
Yes	90 (4.3)	16 (2.8)	74 (4.9)	
Bethesda Category	4.80 ± 1.56	2.66 ± 1.53	4.96 ± 1.44	<0.001
Kwak TIRADS	4.62 ± 1.32	3.31 ± 0.66	5.11 ± 1.15	<0.001
Nodule diameter (cm)	1.8 ± 1.4	3.24 ± 1.6	1.2 ± 0.89	<0.001
TSH (mIU/L)	2.32 ± 1.97	1.95 ± 1.93	2.45 ± 2.02	<0.001
FT3 (pmol/L)	4.94 ± 0.77	4.95 ± 0.74	4.93 ± 0.79	0.629
FT4 (pmol/L)	16.78 ± 3.38	16.82 ± 3.65	16.7 ± 3.30	0.681
TgAb (IU/ml)	11.51 ± 24.08	10.71 ± 6.34	11.9 ± 47.5	<0.001
TPOAb (IU/ml)	17.00 ± 15.00	16.77 ± 11.76	17.1 ± 17.25	0.090
Tg (ng/ml)	20.60 ± 47.30	52.1 ± 178.85	15.7 ± 30.06	<0.001
HDL-C (mmol/L)	1.16 ± 0.43	1.20 ± 0.44	1.15 ± 0.43	0.002
LDL-C (mmol/L)	2.42 ± 0.90	2.45 ± 0.91	2.40 ± 0.90	0.546

Data are expressed as mean ± standard deviation or frequency (%). Kwak TIRADS assignment: 3 assigned to 3, 4a assigned to 4, 4b assigned to 5, 4c assigned to 6, 5 assigned to 7. TIRADS: Thyroid Imaging Reporting and Data Systems. TN, thyroid nodules; PTC, papillary thyroid carcinoma; BMI, body mass index (weight/height2). TSH, thyroid-stimulating hormone; FT3, free triiodothyronine; FT4, free thyroxine; TgAb, antithyroglobulin antibody; TPOAb, anti-thyroid peroxidase antibody; TAb, thyroid autoantibody (positive if TgAb and/or TPOAb are positive); Tg, thyroglobulin; HDL-C, high-density lipoprotein cholesterol; LDL-C, low-density lipoprotein cholesterol.

### 3.2 Logistic regression analysis

Multivariate logistic regression analysis indicated that Bethesda category (OR=1.90, 95% CI 1.62–2.24, P<0.001), TIRADS score (OR=2.55, 95% CI 1.82–3.56, P<0.001), age (OR=0.97, 95% CI 0.94–0.99, P=0.002), nodule size (OR=0.53, 95% CI 0.41–0.69, P<0.001), serum levels of Tg (OR=0.994, 95% CI 0.991–0.998, P=0.004), and HDL-C (OR=0.23, 95% CI 0.10–0.53, P=0.001) were statistically significant independent differentiators for patients with PTC and benign nodules (**Table 2**, **Table S2**).

**Table 2:** Multivariate logistic regression analysis of PTC in TN patients.

Indicator	*B*	*SE*	*Wald*	*df*	*P*	*OR*	95% *CI*	
							Lower limit	Upper limit
Age	-0.036	0.012	9.483	1	0.002	0.965	0.943	0.987
Bethesda Classification	0.643	0.083	59.537	1	<0.001	1.902	1.616	2.240
Family history	1.231	1.248	0.973	1	0.324	3.425	0.297	39.538
History of Radiation	-0.344	1.068	0.104	1	0.747	0.709	0.087	5.753
Maximum diameter	-0.642	0.131	24.091	1	<0.001	0.526	0.407	0.680
Kwak TIRADS	0.935	0.171	29.762	1	<0.001	2.547	1.820	3.564
HDL-C	-1.465	0.425	11.902	1	0.001	0.231	0.101	0.531
Tg	-0.006	0.002	8.457	1	0.004	0.994	0.991	0.998

PTC, papillary thyroid carcinoma; TN, thyroid nodules; Tg, thyroglobulin; HDL-C, high-density lipoprotein cholesterol. TIRADS, Thyroid Imaging Reporting and Data Systems; HDL-C, high-density lipoprotein cholesterol; Tg, thyroglobulin.

**Figure 2:**
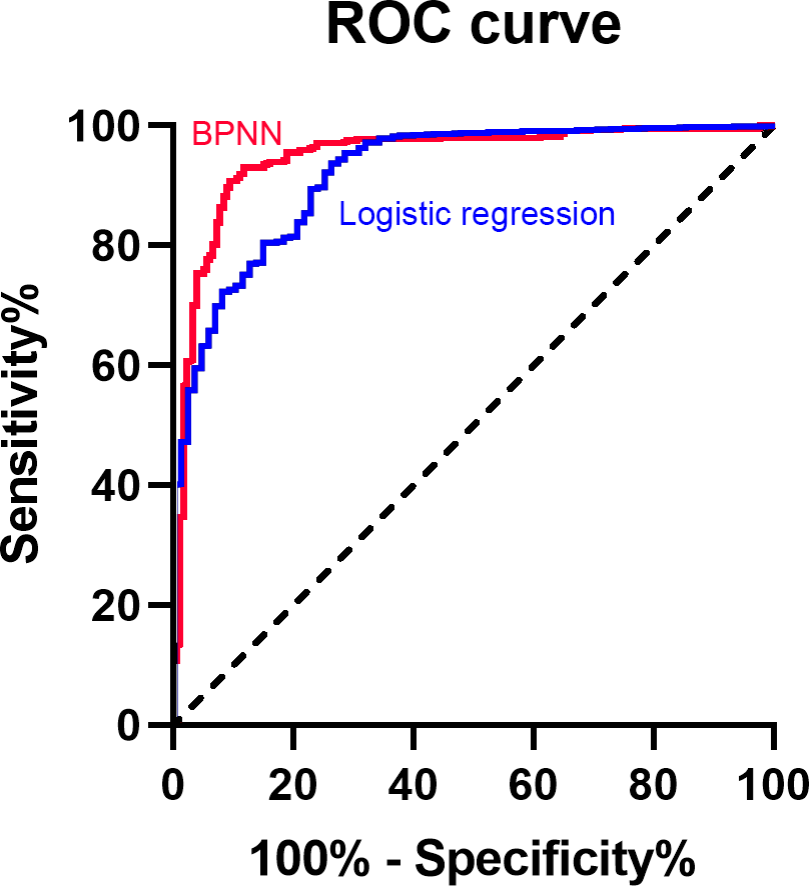
ROC curve of BPNN and multivariate logistic regression model. BPNN, back propagation neural network; ROC, receiver operating characteristic curve.

A multivariate logistic regression model was constructed with the above variables, as well as family history of thyroid tumor and history of radiation exposure, used as the independent variables and the pathological finding as the dependent variable. The corresponding ROC curve is shown in **Figure 2**, and the AUC was 0.924 (95% CI 0.896–0.952, SE=0.014, P<0.001). The Hosmer-Lemeshow goodness-of-fit value was 0.434. The predicted probability (P) value (0.8607) at the highest Youden index (sensitivity+specificity-1) value was selected as the diagnostic cutoff, based on the ROC curve coordinates (sensitivity and 1-specificity) outputted by SPSS. The multivariate logistic regression model had a sensitivity of 93.9%, a specificity of 73.9%, an NPV of 45.8%, and a PPV of 98.1% (**Table 3**).

**Table 3:** Comparison of diagnostic performance of BPNN with multivariate logistic regression model.

Indicator	Multivariate logistic regression	BPNN
AUC	0.924 (0.896-0.952)	0.948 (0.928-0.969)
Sensitivity	93.9% (92.3%-95.1%)	93.1% (90.2%-95.2%)
Specificity	73.9% (63.2%-82.4%)	88.3% (82.5%-92.5%)
Youden index	0.677 (0.555-0.775)	0.814 (0.727-0.877)
NPV	45.8% (37.5%-54.3%)	83.7% (77.5%-88.5%)
PPV	98.1% (97.1%-98.7%)	95.2% (92.6%-96.9%)

AUC, area under receiver operating characteristic curve (95% CI is shown in parentheses); BPNN, back propagation neural network; NPV, negative predictive value; PPV, positive predictive value. Youden index=sensitivity+specificity-1.

### 3.3 BPNN model

Baseline characteristics of the training set and the prediction set are shown in **Table 4**. No significant difference was found between these two groups. Ten indicators, including age, family history of thyroid tumor, history of radiation exposure, nodule size, Bethesda category, TIRADS, serum levels of TSH, TPOAb, Tg, and HDL-C, were selected as the input variables in the BPNN model, i.e., variables with statistical significance in the univariate logistic regression analyses, as well as family history of thyroid tumor and history of radiation exposure, which have notable clinical relevance. The pathological results were set as the output variables. A structural diagram of the model is shown in **Figure S2**.

**Table 4:** Baseline characteristics of TN patients in the training and prediction cohorts.

Indicator	All patients (N=2090)	Training cohort (N=1463)	Prediction cohort (N=627)	*P*
Gender (%)				0.501
Male	546	376 (68.9%)	170 (31.1%)	
Female	1544	1087 (70.4%)	457 (29.6%)	
Age (years)	45.28 ± 13.01	45.98 ± 13.35	44.98 ± 12.86	0.110
BMI (kg/m^2^)	23.83 ± 3.45	23.78 ± 3.42	23.86 ± 3.46	0.615
Nodule type (%)				0.351
Benign	571	391 (68.5%)	180 (31.5%)	
Malignant	1519	1072 (70.6%)	447 (29.4%)	
History of Radiation				0.216
No	2044	1427 (69.8%)	617 (30.2%)	
Yes	46	36 (78.3%)	10 (21.7%)	
Family History				1.000
No	2000	1400 (70.0%)	600 (30.0%)	
Yes	90	63 (70.0%)	27 (30.0%)	
Bethesda Category	4.80 ± 1.56	4.77 ± 1.56	4.81 ± 1.56	0.693
Kwak TIRADS	4.62 ± 1.32	4.67 ± 1.36	4.60 ± 1.30	0.224
Nodule diameter (cm)	1.80 ± 1.40	1.84 ± 1.40	1.79 ± 1.42	0.852
TSH (mIU/L)	2.32 ± 1.97	2.38 ± 2.01	2.24 ± 1.85	0.123
FT3 (pmol/L)	4.94 ± 0.77	4.94 ± 0.78	4.92 ± 0.78	0.904
FT4 (pmol/L)	16.78 ± 3.38	16.74 ± 3.33	16.80 ± 3.40	0.123
TgAb (IU/mL)	11.51 ± 24.08	11.67 ± 25.80	11.30 ± 20.60	0.904
TPOAb (IU/ml)	17.00 ± 15.00	17.00 ± 15.10	17.00 ± 14.75	0.074
Tg (ng/ml)	20.60 ± 47.30	20.60 ± 46.05	20.30 ± 51.32	0.723
HDL-C (mmol/L)	1.16 ± 0.43	1.18 ± 0.43	1.13 ± 0.42	0.630
LDL-C (mmol/L)	2.42 ± 0.90	2.43 ± 0.90	2.41 ± 0.91	0.799

Data are expressed as mean ± standard deviation or frequency (%). Kwak TIRADS assignment: 3 assigned to 3, 4a assigned to 4, 4b assigned to 5, 4c assigned to 6, 5 assigned to 7. TIRADS, Thyroid Imaging Reporting and Data Systems; TN, thyroid nodules; PTC, papillary thyroid carcinoma; BMI, body mass index (weight/height2); TSH, thyroid-stimulating hormone; FT3, free triiodothyronine; FT4, free thyroxine; TgAb, antithyroglobulin antibody; TPOAb, anti-thyroid peroxidase antibody; TAb, thyroid autoantibody (positive if TgAb and/or TPOAb are positive); Tg, thyroglobulin; HDL-C, high-density lipoprotein cholesterol; LDL-C, low-density lipoprotein cholesterol.

After training, the model based on the training set (1463 cases), the results predicted by the BPNN model, and actual results for the 627 patients in the prediction set were compared. The specific values are shown in supplementary file. The AUC was 0.948 (95% CI 0.928–0.969, SE=0.010, P<0.001). A diagnostic cutoff value of 1.5168 (at the highest Youden index value) was selected, with a sensitivity of 93.1%, a specificity of 88.3%, an NPV of 83.7%, and a PPV of 95.2% (**Figure 2**).

As compared with the multivariate logistic regression model, BPNN model showed a comparable but numerically higher AUC (0.948 vs. 0.924; **Table 3**). BPNN model showed a much higher specificity (88.3% vs. 73.9%) than logistic regression model, while their sensitivity (93.1% vs. 93.9%) was similar. BPNN also showed a higher NPV value (83.7% vs. 45.8%) but the PPV value (95.2% vs. 98.1%) was lower.

We also performed stratified analysis based on Bethesda indeterminate cytology categories III to V, and the findings were similar. BPNN model showed a higher specificity (82.3% vs. 77.1%) than logistic regression model, although the AUC was smaller than the overall analysis for both models (**Table S3**).

Inter-operator variability between two sonologists proved to be small by using intraclass correlation coefficients, as provided in **Table S4**.

## 4 Discussion

The assessment of TNs, particularly the risk stratification assessment of TNs by using single features, is not an easy task. A number of studies have applied computer-aided diagnosis (CAD) methodologic analysis to assist the evaluation of TNs. For example, Lee and colleagues developed a CAD system to identify and differentiate metastatic lymph nodes of thyroid cancer. This system proved to be highly sensitive and but relatively less specific for predicting lymph node malignancy (15). A retrospective, multicohort study from China analyzed over 300,000 ultrasound images from patients with thyroid cancer and negative controls, and found that these AI-guided models showed similar sensitivity and improved specificity in identifying thyroid cancer compared with skilled radiologists (10). However, Choi et al. reported that CAD system had a similar sensitivity as experienced radiologist (90.7% vs. 88.4%) but with lower specificity (74.6% vs. 94.9%) (16). Machine learning based on sonographic or pathological images is still under-development and far from mature. For example, the sonographic features may not be adequately captured, or may look differently between axial and transverse images, which might decrease the performance of deep learning (17). Notably, the vast majority of these studies focused on the comparison of radiologists and deep-learning models by reading only ultrasound images. They relied on handcrafted features or deep features extracted from images after processing, where detailed raw information may be distorted or even lost. Besides, these studies inevitably brought intensive labor work to extract features from ultrasound images. In contrast, our study, which was based on a combination of relevant demographic, serological, ultrasound, and biopsy data (all were readily available from a routine clinical practice), showed promising performance in differentiating patients with PTC from benign nodules, and thus might help guide the decision of surgery. To the best of our knowledge, this is the first study that attempted to incorporate different lines of variables, rather than only ultrasound images as used in other studies, into machine-learning model and aimed to aid the diagnosis of PTC.

Results from multivariate logistic regression analysis indicated that Bethesda category and TIRADS were positively associated with PTC, which were in agreement with previous literature, whereas age, nodule size, and serum levels of Tg and HDL-C were negatively associated with PTC. In consistent with our findings, a large prospective study including 6,391 patients showed that the incidence of TNs increased but thyroid malignancy decreased with age (18). Another study involving over 30,000 TN patients undergoing FNAB found that younger age was an independent risk factor for PTC (19). All patients included in our study were all age 18 years or older since the proportion of patients under the age of 18 years, who underwent thyroid surgery in our hospital, were small (most might have received therapy in children’s hospital). Excluding adolescent patients could also prevent the analysis from confounding due to the heterogeneity between adults and adolescents; however, whether our conclusions could be generalized to other age groups needs further investigation.

We included HDL-C and LDL-C in our analysis because some previous studies suggested potential associations between these cholesterol parameters and endocrine-related cancer, including thyroid cancer, although these association was not consistently reported (20). A recent study from China demonstrated that patients with PTC had a higher monocyte to HDL-C ratio (MHR), and MHR was an independent risk factor for PTC (21). It is interesting that in our large study, HDL-C might act as a protective factor for thyroid cancer. Very recently, two studies from South Korea confirmed that a low level of HDL-C was associated with a higher risk of thyroid cancer, especially in a metabolically unhealthy population (22, 23). The mechanism underlying the association between HDL-C and thyroid cancer remains unclear and warrants further investigation. Insulin resistance has been suggested as one of the contributing factors. A decrease of HDL-C often accompanies insulin resistance (24), and diabetes, another condition with insulin resistance, is associated with an increased risk of thyroid cancer (25). It is unclear whether the antioxidant and anti-inflammatory effect of HDL-C was involved (20).

In this study, we found that both BPNN and multivariate logistic regression model had an AUC >0.92, showing a promising performance in differentiating patients with PTC from those with benign nodules. Both models included a variety of variables from different aspects. All of these variables were readily to be obtained from routine clinical practice and were almost noninvasive. It is intriguing that the BPNN model showed a higher diagnostic specificity than the logistic regression model, while the sensitivity was maintained. These specific advantages of BPNN over logistic model make BPNN a more clinically useful approach (26).

FNAB is a routinely recommended pre-operative cytological examination. With the dramatic development of molecular diagnostic technologies in recent years, researchers have discovered *BRAF*, *RAS*, and telomerase reverse transcriptase (*TERT*) promoter mutations, *RET/PTC* rearrangement, and other genetic mutations to be associated with thyroid cancers (27, 28). Several key characteristic molecules such as long noncoding RNAs and microRNAs also show an association (29–31). These genetic technologies may improve the diagnosis for some patients who failed to be diagnosed with cytopathology. We did not include molecular information in our analysis due to limited data, but future studies incorporating molecular data might further increase the accuracy and specificity for prediction of PTC.

With the advance of TNs screening technology and increased detection demand, we can anticipate a dramatic increase of thyroid nodules (32). Thus, the development of a systematic method to differentiate patients with PTC from those with benign nodules is critical, given that there is concern regarding the overdiagnosis and surgery of thyroid cancer. Our study suggests the advantages of combing demographic, serological, and nodule characteristics for the differentiation of PTC from benign nodules. Further prospective studies are warranted to confirm these findings and determine whether similar effect could be generalized to other thyroid tumors. Overtreatment of thyroid tumors is commonly seen in real-world practice in China. If validated, our model might improve the accuracy of defining surgical necessity.

Several limitations should also be acknowledged in our study. First, the retrospective nature of the study made it impossible to avoid potential confounding, although the sonologists and researchers were all well-trained and experienced, and the inter-operator variability between two sonologists was small. Second, all the study subjects were inpatients who underwent thyroid surgery, which might induce selection bias that may favor more complex or malignant cases. We are performing a prospective study to assess the performance of the BPNN and logistic regression models among all FNAB cases with and without surgery to address this bias; and those without surgery will be followed up with ultrasonography. Third, follicular carcinomas were rare in China due to a high iodine supply. Thus, whether our predictive system can be applied to other populations with different iodine supply or to tumors of different histotypes remains to be determined. Fourth, although we utilized a randomly set 30% of patients for validation, an independent validation population is still needed to further validate the performance of our BPNN model. Finally, the BPNN model was divided into two sets, whereas the regression model was evaluated only in one combined set; the difference of patients being compared, although not big, might affect the findings. However, we aimed not to compete with logistic regression model, but to provide a tool with excellent performance in aiding PTC risk stratification.

## 5 Conclusions

Bethesda category, TIRADS, nodule size, and serum levels of HDL-C were most significant differentiators for patients with PTC and those with benign nodules. Based on demographic, serological, ultrasound, and biopsy data, both BPNN and multivariate logistic regression model showed excellent performance in differentiating patients with PTC from those with benign nodules, but the BPNN model provided more specific prediction for PTC than conventional regression analysis, and thus might help guide the decision of surgery. Future studies are needed to validate our findings.

## Data availability statement

The original contributions presented in the study are included in the article/**Supplementary Material**. Further inquiries can be directed to the corresponding authors.

## Ethics statement

The studies involving human participants were reviewed and approved by the Ethics Committee of Nanjing Drum Tower Hospital Institutional Review Board. Written informed consent for participation was not required for this study in accordance with the national legislation and the institutional requirements.

## Author contributions

Conceptualization, XWZ, YB, and DZ; Methodology, XWZ, YZ, and XLZ; Investigation, XWZ, YZ, JS, XS, SS, and XLZ; Writing—original draft preparation, XWZ, YZ, SS, and XLZ; Funding acquisition, XWZ. All authors have read and agreed to the published version of the manuscript.

## Funding

This project was supported by the National Nature Science Funds of China (Grant No. 81800752), and Fundings for Clinical Trials from the Affiliated Drum Tower Hospital, Medical School of Nanjing University (2022-YXZX-NFM-02).

## Conflict of interest

The authors declare that the research was conducted in the absence of any commercial or financial relationships that could be construed as a potential conflict of interest.

## Publisher’s note

All claims expressed in this article are solely those of the authors and do not necessarily represent those of their affiliated organizations, or those of the publisher, the editors and the reviewers. Any product that may be evaluated in this article, or claim that may be made by its manufacturer, is not guaranteed or endorsed by the publisher.
