# Supplementary material for: Risk factors and diagnostic prediction models for papillary thyroid carcinoma
Source: Front Endocrinol (Lausanne). 2022 Sep 5;13:938008. doi: 10.3389/fendo.2022.938008 (PMC9483149; doi:10.3389/fendo.2022.938008)
Supplement: Supplementary file 1 [file Table_1.docx]

**Table S1: AUC of different neural node number**

| **Neural node** | **AUC** | **95%CI LCI** | **95%CI**  **UCI** |
| --- | --- | --- | --- |
| 1 | 0.945 | 0.926 | 0.965 |
| 2 | 0.94 | 0.92 | 0.96 |
| 3 | 0.938 | 0.918 | 0.958 |
| 4 | 0.946 | 0.924 | 0.967 |
| 5 | 0.938 | 0.911 | 0.964 |
| 6 | 0.945 | 0.92 | 0.969 |
| 7 | 0.934 | 0.906 | 0.962 |
| 8 | 0.936 | 0.913 | 0.959 |
| 9 | 0.929 | 0.904 | 0.954 |
| 10 | 0.925 | 0.896 | 0.955 |
| 11 | 0.942 | 0.917 | 0.966 |
| 12 | 0.944 | 0.92 | 0.968 |
| 13 | 0.936 | 0.912 | 0.959 |
| 14 | 0.947 | 0.926 | 0.968 |
| **15** | **0.948** | **0.928** | **0.969** |
| 16 | 0.935 | 0.908 | 0.962 |
| 17 | 0.938 | 0.915 | 0.961 |
| 18 | 0.946 | 0.923 | 0.969 |
| 19 | 0.926 | 0.896 | 0.955 |
| 20 | 0.943 | 0.92 | 0.965 |

**Table S2. Univariate logistic regression analyses of PTC in TN patients**

| **Indicator** | ***OR*** | **95% *CI*** | | ***P*** |
| --- | --- | --- | --- | --- |
|  |  | **Lower limit** | **Upper limit** |  |
| Male sex | 1.263 | 1.008 | 1.581 | 0.043 |
| Age (years) | 0.954 | 0.946 | 0.962 | <0.001 |
| BMI (kg/m2) | 0.998 | 0.971 | 1.026 | 0.892 |
| Bethesda Classification | 2.135 | 1.880 | 2.424 | <0.001 |
| Family History | 1.776 | 1.026 | 3.076 | 0.040 |
| History of Radiation | 3.140 | 1.235 | 7.987 | 0.016 |
| Maximum diameter | 0.314 | 0.282 | 0.349 | <0.001 |
| Kwak TIRADS | 10.795 | 8.673 | 13.436 | <0.001 |
| TSH (mIU/L) | 1.218 | 1.144 | 1.296 | <0.001 |
| FT3 (pmol/L) | 1.005 | 0.928 | 1.089 | 0.903 |
| FT4 (pmol/L) | 0.999 | 0.973 | 1.027 | 0.970 |
| TgAb (IU/mL) | 1.001 | 1.001 | 1.002 | <0.001 |
| TPOAb (IU/mL) | 1.002 | 1.001 | 1.003 | 0.002 |
| Tg (ng/mL) | 0.991 | 0.990 | 0.992 | <0.001 |
| HDL-C (mmol/L) | 0.650 | 0.487 | 0.867 | 0.003 |
| LDL-C (mmol/L) | 0.966 | 0.842 | 1.109 | 0.626 |

PTC, papillary thyroid carcinoma; TN, thyroid nodules; BMI, body mass index (weight/height^2^); TIRADS: Thyroid Imaging Reporting and Data Systems; TSH, thyroid-stimulating hormone; FT3, free triiodothyronine; FT4, free thyroxine; TgAb, antithyroglobulin antibody; TPOAb, anti-thyroid peroxidase antibody; TAb, thyroid autoantibody (positive if TgAb and/or TPOAb are positive); Tg, thyroglobulin; HDL-C, high-density lipoprotein cholesterol; LDL-C, low-density lipoprotein cholesterol.

**Table S3. Comparison of diagnostic performance of BPNN with multivariate logistic regression model for patients with Bethesda categories III-V**

| **Indicator** | **Multivariate logistic regression** | **BPNN** |
| --- | --- | --- |
| AUC (95% CI) | 0.886 (0.843-0.928) | 0.877 (0.817-0.937) |
| Sensitivity (95% CI) | 84.4% (69.9%-93.0%) | 84.3% (70.9%-92.5%) |
| Specificity (95% CI) | 77.1% (74.5%-79.4%) | 82.3% (80.0%-84.4%) |

AUC, area under receiver operating characteristic curve (95% CI is shown in parentheses); BPNN: back propagation neural network.

**Table S4.** Interobserver variability test.

| **Variables** | **Intraclass correlation coefficient** | **95% confidence interval** | **P value** |
| --- | --- | --- | --- |
| TIRADS score | 0.832 | 0.348 to 0.958 | <0.001 |
| Nodule size (cm) | 0.908 | 0.691 to 0.976 | <0.001 |


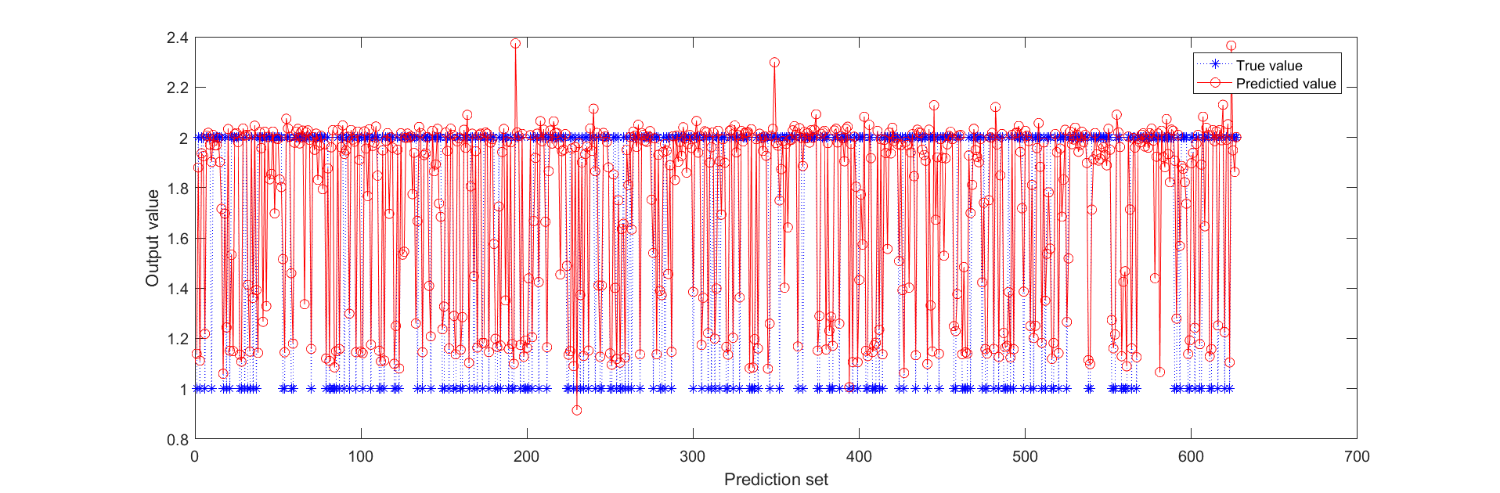


**Figure S1.** Comparison of actual and predicted values of the prediction set. 1.000=benign TN; 2.000=PTC. TN, thyroid nodules; PTC, papillary thyroid carcinoma.


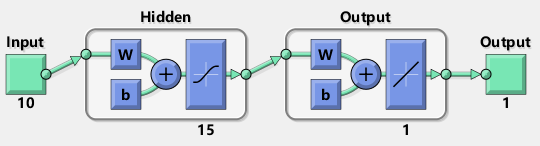


**Figure S2.** Structure of BPNN model. BPNN, back propagation neural network.

**Software Code**

% Randomly generate training set and prediction set

temp = randperm(size(Y,1));

% Training data set (1463)

P_train = Y(temp(1: 1463),:)';

T_train = Z(temp(1: 1463,:)';

% Prediction data set (the rest)

P_test = Y(temp(1464:end),:)';

T_test = Z(temp(1464:end),:)';

N = size(P_test,2);

%Data Normalization

[p_train, ps_input] = mapminmax(P_train, 0, 1);

p_test = mapminmax('apply', P_test,ps_input);

[t_train, ps_output] = mapminmax(T_train, 0, 1);

% Network construction

>> net=feedforwardnet(15,'trainlm');

% Parameter setting

>> net.trainParam.epochs = 10000;

net.trainParam.goal = 1e-5;

net.trainParam.lr = 0.01;

%Running

>> net = train(net,p_train,t_train);

>> view(net)

>> net

%Inverse normalization

>> t_sim = sim(net,p_test);

>> T_sim = mapminmax('reverse',t_sim,ps_output);

%Error output and R2 calculation

>> error = abs(T_sim - T_test)./T_test;

>> R2 = (N * sum(T_sim .* T_test) - sum(T_sim) * sum(T_test))^2 / ((N * sum((T_sim).^2) - (sum(T_sim))^2) * (N * sum((T_test).^2) - (sum(T_test))^2));

>> result = [T_test' T_sim' error']

% Plotting

>> figure

plot(1:N,T_test,'b:*',1:N,T_sim,'r-o')

legend('True value','Predictied value')

xlabel('Prediction set')

ylabel('Output value')

string = {'The comparison between the predicted value and the true value ';['R^2=' num2str(R2)]};

**Comparison between predicted and actual values**

| **Actual** | **Predicted** | **Actual** | **Predicted** | **Actual** | **Predicted** | **Actual** | **Predicted** | **Actual** | **Predicted** |
| --- | --- | --- | --- | --- | --- | --- | --- | --- | --- |

2 2.0333

1 1.1631

1 1.4392

2 1.351

2 2.0086

2 1.9415

2 1.9834

1 1.0994

1 1.1921

1 1.2048

2 2.0144

1 1.158

1 1.1738

2 2.3737

1 1.4243

2 1.9799

2 2.0137

2 1.6668

2 2.0192

2 2.0647

1 1.1718

2 1.918

1 1.1781

2 2.0052

1 1.1275

2 1.9848

2 2.0302

2 1.7247

2 2.0271

2 1.6641

2 1.9754

1 1.165

2 2.0636

1 1.166

2 1.8658

2 2.0176

2 2.0173

1 1.1987

2 1.9727

2 2.0128

2 1.9793

2 1.4544

2 1.9925

2 1.9452

1 1.4891

2 2.0144

2 1.5762

2 1.9508

1 1.1351

1 1.1459

2 1.1495

2 1.1824

2 1.956

2 2.019

1 1.0901

1 1.1822

2 1.9614

1 0.9141

2 2.0145

2 1.9772

2 1.9331

2 2.0299

1 1.3728

2 1.963

2 1.9581

2 1.9005

2 2.0895

2 2.0092

1 1.1518

1 1.1305

1 1.2848

1 1.1032

2 2.0353

2 1.9454

2 1.9463

2 1.805

1 1.1637

2 2.1137

1 1.1549

2 2.0146

1 1.8652

1 1.4471

2 2.0177

1 1.29

2 1.9962

2 1.4108

1 1.1272

1 1.1368

1 1.4105

2 1.984

2 2.0104

2 2.0175

2 2.0031

2 1.9821

2 2.0342

2 1.8802

2 1.9458

2 1.4009

1 1.1406

2 2.0338

1 1.1595

1 1.1182

1 1.0956

1 1.327

2 1.7369

2 1.8526

2 1.7495

2 2.019

1 1.104

2 1.8925

2 1.6837

1 1.6357

1 1.2365

2 1.6564

2 2.0015

2 1.8649

1 1.124

1 1.6333

2 1.9621

2 1.9502

2 2.0233

2 2.05

1 1.81

1 1.1367

2 2.0069

2 2.0263

2 1.93

2 2.017

1 1.3895

2 2.0094

2 2.0248

1 1.2083

2 1.1369

1 1.3726

2 1.9863

2 1.7518

2 1.9418

2 1.9818

1 1.5398

1 1.9725

2 2.0046

2 1.409

2 1.9959

2 1.9469

2 1.4563

2 2.0007

2 1.8891

1 1.1469

2 2.0045

2 1.9361

2 1.8303

1 1.1454

2 2.0067

2 1.9294

2 1.9028

2 2.0414

2 1.9817

2 2.017

2 1.9259

1 1.6672

2 2.04

2 1.9949

2 1.8595

2 1.2596

2 1.9735

2 1.7739

2 2.0092

2 1.9578

2 1.9382

1 1.3853

2 2.0036

2 2.0056

2 1.9965

1 1.1743

2 2.0651

2 1.362

2 1.9996

2 1.9391

2 2.0241

2 2.0201

2 2.015

1 1.2211

2 1.8998

1 1.9607

2 1.998

2 2.0201

1 1.1991

2 1.5452

2 1.3988

2 2.0263

2 1.5335

1 1.9054

2 1.6926

2 2.0161

2 1.9925

2 1.8989

1 1.1663

1 1.0805

1 1.1353

2 2.0257

2 1.9508

2 2.0305

1 1.2028

1 1.2498

2 2.0468

2 1.9403

1 1.0991

2 2.0088

1 1.3629

2 2.025

2 1.9591

2 1.9832

2 1.9997

2 2.0177

2 2.0193

2 2.0206

2 1.6959

1 1.0815

1 2.0324

2 2.0165

1 1.0842

1 1.1968

2 1.987

2 1.9925

2 1.9265

1 1.1602

2 1.0794

2 2.0144

1 1.2589

1 1.112

2 1.9968

2 2.0042

2 2.0067

2 1.9456

2 2.0336

2 2.2984

2 1.948

2 1.9771

2 1.9666

1 1.1089

1 1.7489

2 1.8733

2 1.9847

1 1.1509

2 1.4019

2 1.9742

2 1.8475

2 1.6416

2 1.9884

2 1.9901

2 2.0431

2 2.01

2 2.0278

1 1.1684

2 2.0444

2 2.0367

2 1.9788

2 1.9978

2 1.966

1 1.8861

2 2.032

2 2.0217

1 1.1753

2 1.9964

2 2.0168

2 1.7663

2 1.9789

2 2.028

2 1.955

2 1.9981

2 2.0302

2 2.0917

2 2.0234

1 1.1516

1 1.2895

2 2.014

1 1.1415

2 2.02

2 2.0259

2 1.1482

2 1.1563

2 1.977

2 1.9101

1 1.2308

1 1.2861

1 1.1707

2 2.0231

2 2.0269

2 2.0341

1 1.145

2 1.9795

2 1.9827

1 1.2587

2 2.0041

2 1.9963

2 1.9911

2 1.9048

2 2.0335

2 2.0421

2 2.0293

1 1.0061

2 1.9738

1 1.2988

1 1.1061

2 2.0027

2 1.8033

2 2.0028

1 1.1053

2 1.433

2 1.9483

2 1.7725

2 1.5735

1 1.9345

2 2.0817

1 1.151

2 2.0472

1 1.1297

2 2.0497

2 1.9604

2 1.9173

1 1.1577

1 1.1458

2 2.0294

1 1.1724

1 1.1832

2 2.0256

1 1.1492

1 1.2344

2 1.9924

1 1.0851

1 1.1369

2 1.9897

1 2.0287

2 1.9375

2 1.5561

1 1.9604

2 2.0184

2 1.936

2 2.0379

2 1.9851

2 1.9956

1 1.1102

2 1.9725

1 1.5079

2 1.9693

2 1.3926

1 1.0629

2 1.8763

2 1.9827

2 1.9691

1 1.1202

2 1.4021

2 1.9397

2 1.9723

2 2.0124

2 1.8452

2 2.0063

1 1.1333

2 1.7935

2 2.0304

2 2.0191

2 1.9671

2 2.0177

2 1.951

2 1.8307

2 1.9299

2 1.9068

2 2.0148

1 1.0984

2 2.0305

2 1.3314

2 1.9506

2 1.1483

2 2.1276

2 2.0278

2 2.0005

2 1.6719

2 2.0266

2 1.9206

2 1.9901

2 1.3363

1 1.1392

1 1.1585

2 2.0194

2 1.9928

2 1.9165

2 1.9994

2 1.9722

2 1.9189

2 1.9757

2 1.529

2 2.0059

2 2.0217

2 2.0337

2 1.9928

2 2.0231

1 1.2489

1 1.2293

2 1.3767

2 1.9786

2 2.0073

1 1.1794

2 2.0097

1 1.1373

1 1.4596

1 1.4838

1 1.1449

2 2.0355

1 1.1398

2 2.0022

2 1.9282

1 1.699

2 1.811

2 2.0739

2 2.0339

2 1.9495

1 1.1446

2 1.9225

2 1.9009

2 1.9934

1 1.5154

2 1.8021

1 1.4229

2 1.74

1 1.1576

1 1.1405

2 1.7497

2 1.8322

2 1.9997

2 2.0174

1 1.1585

2 1.9934

2 2.1205

2 1.9719

2 1.9943

1 1.1258

2 1.6972

2 1.8488

2 2.0131

2 2.0232

1 1.2219

2 1.8543

1 1.1643

1 1.1708

2 1.3848

2 1.8332

1 1.1242

2 2.0018

2 2.0098

1 1.1569

2 1.3284

2 2.0166

2 2.0188

2 2.0223

2 2.045

2 1.9418

2 1.266

2 1.7177

1 1.3863

2 2.0254

2 1.957

2 2.0061

2 1.9854

2 2.021

1 1.2497

2 1.8103

2 1.1425

1 1.2017

1 1.3924

2 1.2505

2 1.9571

2 2.0463

2 2.0565

1 1.3617

2 1.8824

1 1.1828

2 2.0114

2 1.9797

1 1.3503

1 1.1479

2 1.5367

2 1.781

2 1.5573

1 1.4132

1 1.1191

1 1.1819

2 2.0093

2 2.0128

2 1.9408

1 1.1415

1 2.007

2 1.9555

2 2.0346

2 1.6843

2 1.832

1 1.1077

2 2.0299

1 1.2657

1 1.1357

2 1.5181

2 1.9913

2 2.0231

2 1.9812

2 2.0388

2 1.9729

2 2.0096

2 2.017

2 1.9424

2 2.0138

2 1.9699

2 2.0224

1 1.114

2 1.1498

2 1.9816

1 1.0981

2 2.0092

2 1.7124

2 1.533

2 1.9124

2 1.8981

2 2.0169

2 1.9325

1 1.1515

2 1.9075

2 2.033

2 1.9155

2 1.9738

2 1.9601

1 1.2438

2 1.9303

2 1.8886

2 1.6969

2 2.0338

2 1.9505

1 1.2734

1 1.0596

1 1.1606

2 1.7157

1 1.2158

2 2.0901

2 2.0195

2 1.9021

2 1.9612

1 1.1292

2 1.9977

1 1.4255

1 1.4673

2 1.9686

1 1.0892

2 2.0032

2 1.7132

2 1.9678

1 1.1599

2 1.9976

2 2.0095

2 1.9594

1 1.1253

2 1.9938

1 1.9017

2 1.9796

2 2.0107

2 1.9707

2 2.016

2 1.9636

2 1.9941

2 2.0181

2 1.9254

2 1.9589

1 1.2184

2 2.0164

2 2.0065

2 2.0343

2 1.9365

2 1.9869

2 1.4397

1 1.1116

2 1.923

2 2.0153

2 1.066

2 1.88

2 2.0263

2 1.9214

1 1.1392

2 1.8814

2 2.0724

2 1.9334

2 1.8213

2 2.0308

2 2.026

1 1.9066

1 1.2788

2 2.0101

1 1.568

2 1.8862

2 1.8749

2 1.8217

2 1.736

1 1.1381

1 1.1923

2 1.8968

2 1.9387

1 1.242

2 1.9743

2 2.0097

1 1.1773

1 1.8915

2 2.0824

2 1.6459

2 2.0379

2 2.0086

1 1.1285

1 1.158

2 2.0285

2 1.985

2 2.0237

1 1.2514

2 2.0287

2 1.9885

2 2.1292

1 1.2252

2 1.9893

2 2.0522

1 1.1048

2 2.3653

2 1.9483

2 1.8625

2 2.0009

1.000=benign TN; 2.000=PTC. TN, thyroid nodules; PTC, papillary thyroid carcinoma.
